# Ursolic Acid Inhibits Na^+^/K^+^-ATPase Activity and Prevents TNF-α-Induced Gene Expression by Blocking Amino Acid Transport and Cellular Protein Synthesis

**DOI:** 10.3390/biom1010032

**Published:** 2011-11-07

**Authors:** Tomonobu Yokomichi, Kyoko Morimoto, Nana Oshima, Yuriko Yamada, Liwei Fu, Shigeru Taketani, Masayoshi Ando, Takao Kataoka

**Affiliations:** 1 Department of Applied Biology, Kyoto Institute of Technology, Matsugasaki, Sakyo-ku, Kyoto 606-8585, Japan; E-Mails: yokkon424@yahoo.co.jp (T.Y.); morikyon-073128@m2.gyao.ne.jp (K.M.); yamada239@sekisui.jp (Y.Y.); taketani@kit.ac.jp (S.T.); 2 Center for Biological Resources and Informatics, Tokyo Institute of Technology, 4259 Nagatsuta-cho, Midori-ku, Yokohama 226-8501, Japan; E-Mail: nana.ooshima@to.shiseido.co.jp (N.O.); 3 Graduate School of Science and Technology, Niigata University, Igarashi 2-8501, Nishi-ku, Niigata 950-2181, Japan; E-Mail: fu-liwei@mgc.co.jp (L.F.); 4 Department of Chemistry and Chemical Engineering, Niigata University, Ikarashi 2-8501, Nishi-ku, Niigata 950-2181, Japan; E-Mail: andomasa2@ksj.biglobe.ne.jp (M.A.)

**Keywords:** amino acid transports, intercellular adhesion molecule-1, Na^+^, K^+^-ATPase, nuclear factor-κB, protein synthesis, ursolic acid, tumor necrosis factor-α

## Abstract

Pro-inflammatory cytokines, such as tumor necrosis factor (TNF)-α, induce the expression of a wide variety of genes, including intercellular adhesion molecule-1 (ICAM-1). Ursolic acid (3*β*-hydroxy-urs-12-en-28-oic acid) was identified to inhibit the cell-surface ICAM-1 expression induced by pro-inflammatory cytokines in human lung carcinoma A549 cells. Ursolic acid was found to inhibit the TNF-α-induced ICAM-1 protein expression almost completely, whereas the TNF-α-induced ICAM-1 mRNA expression and NF-κB signaling pathway were decreased only partially by ursolic acid. In line with these findings, ursolic acid prevented cellular protein synthesis as well as amino acid uptake, but did not obviously affect nucleoside uptake and the subsequent DNA/RNA syntheses. This inhibitory profile of ursolic acid was similar to that of the Na^+^/K^+^-ATPase inhibitor, ouabain, but not the translation inhibitor, cycloheximide. Consistent with this notion, ursolic acid was found to inhibit the catalytic activity of Na^+^/K^+^-ATPase. Thus, our present study reveals a novel molecular mechanism in which ursolic acid inhibits Na^+^/K^+^-ATPase activity and prevents the TNF-α-induced gene expression by blocking amino acid transport and cellular protein synthesis.

## 1. Introduction

Pro-inflammatory cytokines, such as tumor necrosis factor (TNF)-α, induce the expression of a variety of genes essential for inflammatory responses, including intercellular adhesion molecule-1 (ICAM-1; CD54) [1]. In response to pro-inflammatory cytokines, ICAM-1 expression is induced on the surface of vascular endothelial cells and required for tissue infiltration of circulating leukocytes [2]. Thus, ICAM-1 plays a critical role in inflammatory response and its expression and function are regarded as a potential target of therapeutic intervention. ICAM-1 expression is regulated mainly at the transcription level and induced by the transcription factor nuclear factor κB (NF-κB) [3].

The NF-κB signaling pathway is induced by various stimuli, including pro-inflammatory cytokines, infectious agents, and chemical/physical stress. The NF-κB dimer is sequestered in the cytosol by associating with the inhibitor of NF-κB (IκB) family of proteins [4]. Upon TNF-α stimulation, TNF receptor 1 triggers the activation of IκB kinase and phosphorylated IκB undergoes ubiquitination and subsequent hydrolysis by the proteasome [5]. The NF-κB dimer then translocates from the cytosol to the nucleus where it activates the transcription of responsive genes that regulate inflammation, proliferation, cell death, cell survival, and angiogenesis [6]. Many types of natural and synthetic small molecules have been reported to block the NF-κB signaling pathway and its downstream gene expression [7].

**Figure 1:**
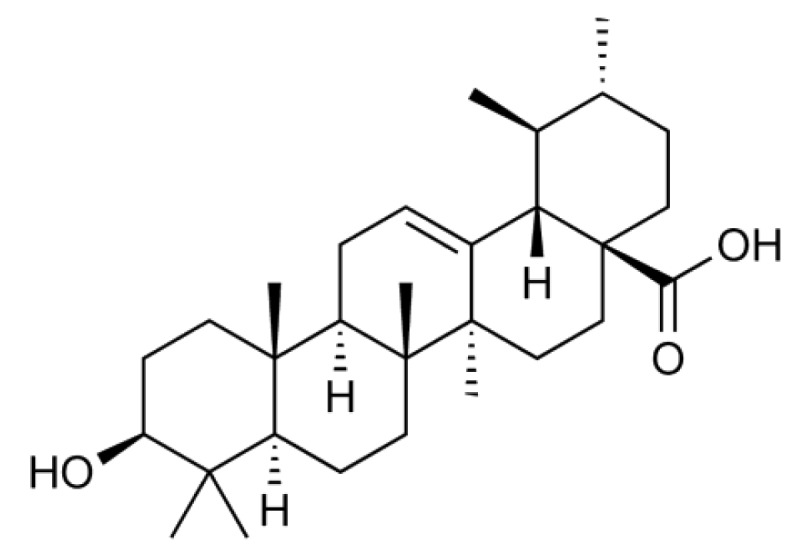
Structure of ursolic acid.

Ursolic acid (3*β*-hydroxy-urs-12-en-28-oic acid) (Figure 1) is a natural pentacyclic triterpenoid that is often found as a major component of medicinal herbs, foods, and other plants. It has been reported that ursolic acid possesses a wide range of biological activities, such as anti-inflammatory and anti-carcinogenic activities [8]. In a screening for anti-inflammatory agents, we were able to isolate and investigate many plant-derived natural compounds. We found that ursolic acid most effectively inhibits the inducible expression of cell-surface ICAM-1 among ursane-, oleanane-, lupane-, and taraxasterane-type triterpenes purified from *Nerium oleander* [9,10]. In relation to this finding, it has been thus far reported that ursolic acid inhibits the NF-κB-dependent signaling pathway and gene expression [11,12,13,14,15,16,17,18]. However, in contrast to these reports, we unexpectedly found that ursolic acid inhibits ICAM-1 expression more effectively by targeting intracellular processes downstream of mRNA expression. In this study, the molecular mechanism by which ursolic acid inhibits ICAM-1 expression was investigated.

## 2. Experimental Section

### 2.1. Cell Culture

Human lung carcinoma A549 cells (JCRB0076) and human fibrosarcoma HT-1080 cells (JCRB9113) were provided by Health Science Research Resources Bank (Osaka, Japan). A549 cells, HT-1080 cells and human breast adenocarcinoma MCF7 cells were maintained in RPMI 1640 medium (Invitrogen, Carlsbad, CA, USA) supplemented with 10% (v/v) heat-inactivated fetal calf serum (JRH Biosciences, Lenexa, KS, USA) and penicillin-streptomycin mixed solution.

### 2.2. Reagents

Ursolic acid was prepared from the leaves of *Nerium oleander* as described previously [9] or commercially purchased from Sigma-Aldrich (St. Louis, MO, USA). Cycloheximide, MG-132 [Z-Leu-Leu-Leu-H (aldehyde)], and ouabain were obtained from Wako Pure Chemical Industries (Osaka, Japan), Peptide Institute (Osaka, Japan), and Sigma-Aldrich, respectively. Antibodies to β-actin (AC-15; Sigma-Aldrich), c-FLIP (Dave-II; Alexis, Lausen, Switzerland), ICAM-1 (clone 15.2; Leinco Technologies, St. Louis, MO, USA), ICAM-1 (clone 28; BD Biosciences, Franklin Lakes, NJ, USA), IκBα (clone 25; BD Biosciences), p65 (20/NF-kB/p65; BD Biosciences) and horseradish-peroxidase (HRP)-linked anti-mouse and anti-rat IgG antibodies (Jackson Immunoresearch, West Grove, PA, USA) were commercially obtained.

### 2.3. Assay for Cell-Surface ICAM-1 Expression

A549 cells were washed twice with phosphate-buffered saline (PBS) and fixed with 1% paraformaldehyde–PBS for 15 min. After washing twice with PBS, the cells were incubated with 1% bovine serum albumin–PBS overnight. Fixed cells were treated with mouse anti-ICAM-1 IgG antibody (clone 15.2) for 60 min and then washed three times with 0.02% Tween 20–PBS. The cells were further treated with HRP-linked anti-mouse IgG antibody for 60 min and then washed three times with 0.02% Tween-20–PBS. To develop the colorimetric reaction, the cells were incubated with the substrate solution (0.2 M sodium citrate (pH 5.3), 0.1% *o*-phenylenediamine dihydrochloride, 0.02% H_2_O_2_) for 20 min at 37 °C. Absorbance at 415 nm was measured with a Model 680 microplate reader (Bio-Rad Laboratories, Hercules, CA, USA).

### 2.4. Assay for Cell Viability

A549 cells were pulsed with 3-(4,5-dimethylthiazol-2-yl)-2,5-diphenyl tetrazolium bromide (MTT; 500 μg/mL) for 4 h and resultant MTT formazan was solubilized with 5% sodium dodecyl sulfate overnight. Absorbance at 595 nm was measured with a Model 680 microplate reader.

### 2.5. Western Blotting

A549 cells, HT-1080 cells and MCF7 cells were harvested with a cell scraper, washed once with PBS, and lysed with Triton X-100 lysis buffer consisting of 50 mM Tris-HCl (pH 7.4), 1% Triton X-100, 2 mM dithiothreitol, 2 mM sodium orthovanadate, and the protease inhibitor cocktail Complete™ (Roche Diagnostics, Mannheim, Germany). Postnuclear lysate was prepared by centrifugation (10,000 × *g*, 5 min). Protein samples (30 μg/lane) were separated by SDS-PAGE and transferred onto Hybond-ECL nitrocellulose membranes (GE Healthcare, Piscataway, NJ, USA). The membranes were incubated with primary antibodies and then HRP-conjugated secondary antibodies, followed by analysis using ECL Western blotting detection reagents (GE Healthcare).

### 2.6. Quantitative RT-PCR

Total RNA was extracted from A549 cells using Sepasol^®^-RNA I super (Nacalai Tesque, Kyoto, Japan) and reverse-transcribed to cDNA using ReverTra Ace^®^ (Toyobo, Osaka, Japan) and oligo(dT)_20_ according to the manufacturer's instructions. cDNA was amplified with a 7000 Fast Real-Time PCR System (Applied Biosystems, Foster City, CA, USA) using SYBR^®^ Premix Ex Taq™ (Takara Bio, Otsu, Japan) and KOD -Plus- Ver. 2 DNA polymerase (Toyobo). The following primer pairs were used: ICAM-1: 5′-GGGAGGCTCCGTGCTGGTGA-3′ (forward) and 5′-TCAGTGCGGCACGAGA AATTG-3′ (reverse) [19]; c-FLIP_L_: 5′-GCCTGTATGCCCGAGCACCG-3′ (forward) and 5′-GCAGG GGGAGCCCTGAGTGA-3′ (reverse) [20]; β-actin: 5′-GGCATCGTGATGGACTCCG-3′ (forward) and 5′-GCTGGAAGGTGGACAGCGA-3′ (reverse). PCR conditions were 94 °C for 3 min, followed by 40 cycles of 94 °C for 15 s, 58 °C for 30 s, and 68 °C for 1 min. The quantity of the initial cDNA was calculated from primer-specific standard curves. The expression level of ICAM-1 and c-FLIP_L_ was normalized on the basis of β-actin levels.

### 2.7. Reporter Assay

A549 cells were transfected with a plasmid encoding κB-responsive firefly luciferase reporter gene using the Lipofectamine™ 2000 transfection reagent (Invitrogen) for 24 h. The transfected A549 cells were treated with agents as indicated. The cells were washed once with PBS, lysed with Triton X-100 lysis buffer, and centrifuged (10,000 × *g*, 5 min). Equal amounts of cell lysates were mixed with luciferase assay solution (0.25 mM luciferin, 0.8 mM ATP, 1 mM dithiothreitol, 9 mM MgCl_2_, 25 mM Tris-phosphate (pH 7.8), 15% glycerol) and relative light units were immediately measured with a Lumitester K-100 Luminometer (Hamamatsu Photonics, Hamamatsu, Japan).

### 2.8. Assay for DNA, RNA and Protein Syntheses

A549 cells were pulse-labeled with [^3^H]L -leucine (41.66 TBq/mmol; Moravek Biochemicals, Brea, CA, USA), [^3^H]L -glutamine (1.628 TBq/mmol; American Radiolabeled Chemicals, St. Louis, MO, USA), [^3^H]thymidine (2.37 TBq/mmol; MP Biomedicals, Santa Ana, CA, USA), and [^3^H]uridine (0.626 TBq/mmol; Moravek) for the indicated times. The labeled cells were washed three times with PBS and then lysed with 250 mM NaOH for 15 min, followed by 1 h incubation on ice in the presence of 5% trichloroacetic acid. The precipitates and the supernatants were separated by centrifugation (10,000 × g, 5 min). Radioactivity was measured with a 1900CA TRI-CARB^®^ liquid scintillation analyzer (Packard Instrument, Meriden, CT, USA).

### 2.9. Assay for Cell-Free Protein Synthesis

Luciferase control RNA was subjected to the cell-free reaction (30 °C, 90 min) for translation by the Rabbit Reticulocyte Lysate System (Promega, Madison, WI, USA). Reaction mixtures were mixed with the luciferase assay solution and relative light units were immediately measured with the Lumitester K-100 Luminometer.

### 2.10. Assay for Amino Acid Transport

A549 cells were treated with agents as indicated in control incubation buffer (125 mM NaCl, 4.8 mM KCl, 1.3 mM CaCl_2_, 1.2 mM MgSO_4_, 25 mM Hepes, 1.2 mM KH_2_PO_4_, 5.6 mM glucose, pH 7.4) and pulse-labeled with [^3^H]L -serine (0.74 TBq/mmol; Moravek) for 5 min. This was followed by the addition of cold L-serine at the final concentration of 500 μM. The cells were immediately washed three times with ice-cold PBS and lysed with 250 mM NaOH, and this was followed by radioactivity measurement.

### 2.11. Assay for Na^+^/K^+^-ATPase Activity

Highly purified Na^+^/K^+^-ATPase from porcine kidney (15.6 μmol Pi/min/mg protein at 37 °C) was a generous gift from Drs. Yoshikazu Tahara and Yutaro Hayashi (Department of Biochemistry, Kyorin University School of Medicine, Tokyo, Japan) [21,22] Assay conditions for the ATP-hydrolyzing activity of Na^+^/K^+^-ATPase were described previously [23]. The colorimetric assay for inorganic phosphate was basically performed as described previously [24].

### 2.12. Statistical Analysis

Statistical significance was assessed by one-way ANOVA followed by the Tukey test for multiple comparisons. Differences of *P* <0.05 were considered to be statistically significant.

## 3. Results and Discussion

### 3.1. Ursolic Acid Inhibits TNF-α-induced ICAM-1 Expression at Intracellular Processes Downstream of mRNA Expression

Human lung carcinoma A549 cells were alveolar epithelial cells highly responsive to pro-inflammatory cytokines and induced to express cell-surface ICAM-1, which was easily measured by the Cell ELISA assay. A549 cells were thus used as a model cell line for screening for anti-inflammatory agents as well as elucidation of their mode of actions. In our previous studies [9,10], ursolic acid was initially identified to inhibit the cell-surface ICAM-1 expression induced by pro-inflammatory cytokines.

**Figure 2:**
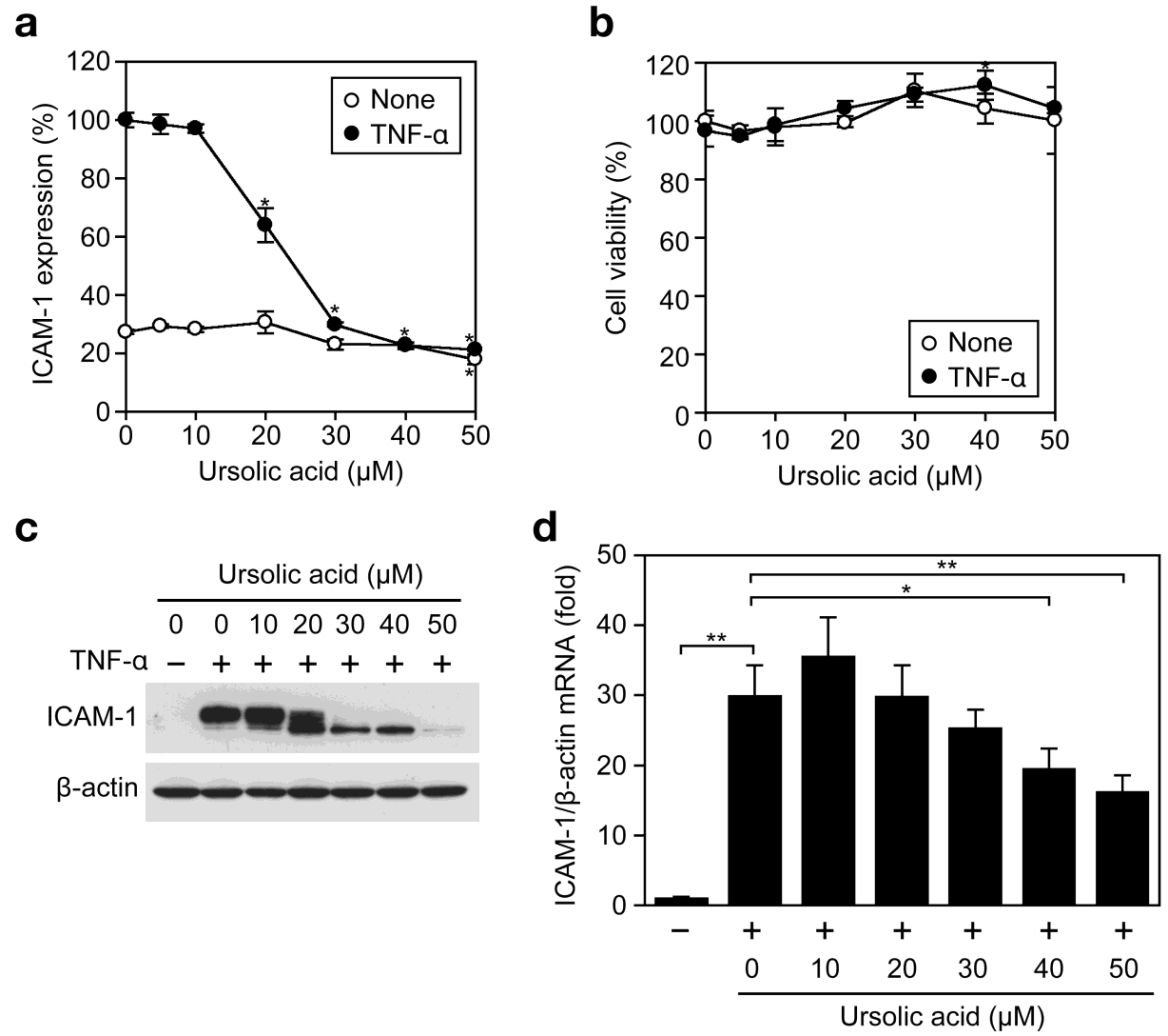
Ursolic acid inhibits TNF-α-induced ICAM-1 expression. (**a**) A549 cells were preincubated with various concentrations of ursolic acid for 1 h and then incubated with (filled circles) or without (open circles) TNF-α (2.5 ng/mL) for 6 h in the presence of ursolic acid. ICAM-1 expression (%) was measured by the Cell ELISA assay. Data points represent means ± SD (n = 3). * *P* < 0.01, compared with control; (**b**) A549 cells were incubated with various concentrations of ursolic acid for 1 h and then incubated with (filled circles) or without (open circles) TNF-α (2.5 ng/mL) for 6 h in the presence of ursolic acid. Cell viability (%) was measured by the MTT assay. Data points represent means ± SD (n = 3). * *P* < 0.05, compared with control; (**c**) A549 cells were preincubated with various concentrations of ursolic acid for 1 h and then incubated with (+) or without (–) TNF-α (2.5 ng/mL) for 6 h. Protein expression of ICAM-1 was analyzed by Western blotting; (**d**) A549 cells were pretreated with indicated concentrations of ursolic acid for 1 h and then incubated with (+) or without (–) TNF-α (2.5 ng/mL) for 6 h in the presence of ursolic acid. ICAM-1 mRNA expression was measured by quantitative RT-PCR. The ratio of ICAM-1 mRNA relative to β-actin mRNA is shown as means ± SD (n = 3). * *P* < 0.05 and ** *P* < 0.01, compared with control.

A549 cells were preincubated with various concentrations of ursolic acid for 1 h and then incubated with TNF-α for 6 h in the presence of ursolic acid. Ursolic acid inhibited the TNF-α-induced expression of cell-surface ICAM-1 in a dose-dependent manner and to the background level at concentrations higher than 30 μM (Figure 2a). This was not caused by the induction of cell death, since ursolic acid up to 50 μM did not decrease cell viability in the presence or absence of TNF-α during the same incubation time (Figure 2b). The amount of total ICAM-1 protein as translation product was then analyzed by Western blotting. Ursolic acid inhibited the TNF-α-induced ICAM-1 expression profoundly at concentrations higher than 30 μM (Figure 2c). In ursolic acid-treated samples, ICAM-1 protein bands migrated faster than those in control samples (Figure 2c), suggesting the ursolic acid may influence post-translational modification. To further investigate the effect of ursolic acid on ICAM-1 transcription, mRNA levels were evaluated by quantitative RT-PCR. TNF-α induced a drastic induction of ICAM-1 mRNA (Figure 2d). However, ursolic acid did not inhibit the TNF-α-induced ICAM-1 mRNA expression at concentrations up to 30 μM and decreased it partially at 40-50 μM (Figure 2d), although ICAM-1 expression at the cell-surface level as well as the protein level was almost completely inhibited by ursolic acid (Figure 2a and Figure 2c). These data suggest that ursolic acid inhibits the TNF-α-induced ICAM-1 expression more effectively at processes downstream of ICAM-1 transcription.

**Figure 3:**
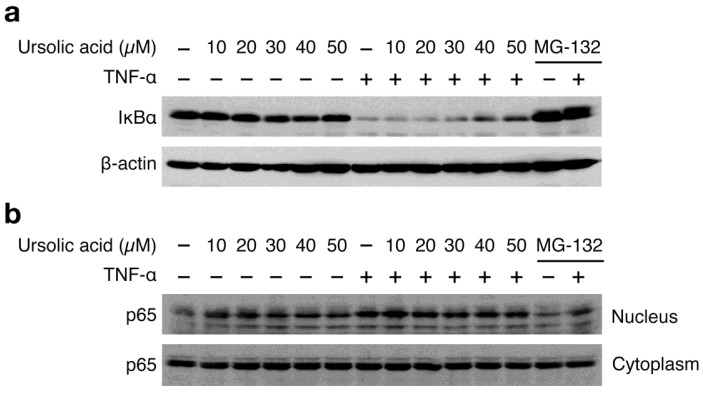
Ursolic acid does not efficiently inhibit TNF-α-induced NF-κB signaling pathway. (**a**) A549 cells were preincubated with indicated concentrations of ursolic acid or MG-132 (20 μM) or without any of those compounds (–) for 1 h and then incubated with (+) or without (–) TNF-α (2.5 ng/mL) for 15 min in the presence of those compounds. Cell lysates were analyzed by Western blotting; (**b**) A549 cells were preincubated with indicated concentrations of ursolic acid or MG-132 (20 μM) or without any of those compounds (–) for 1 h and then incubated with (+) or without (–) TNF-α (2.5 ng/mL) for 30 min in the presence of those compounds. Cell lysates were analyzed by Western blotting.

It has been shown that TNF-α-induced ICAM-1 expression is highly NF-κB-dependent in A549 cells [25]. TNF-α stimulation induced the drastic degradation of IκBα (Figure 3a) and the nuclear translocation of the NF-κB subunit p65 (Figure 3b). Ursolic acid weakly affected the TNF-α-induced IκBα degradation in A549 cells, as compared with the proteasome inhibitor MG-132 that markedly prevented the IκBα degradation (Figure 3a). In addition, unlike MG-132, ursolic acid only marginally decreased the TNF-α-induced p65 translocation to the nucleus (Figure 3b). In the absence of TNF-α stimulation, it seems that ursolic acid alone increases p65 nuclear translocation to some extent without inducing IκBα degradation (Figure 3a and Figure 3b). Consistent with a partial reduction of ICAM-1 mRNA expression by ursolic acid (Figure 2d), these data indicate that ursolic acid does not efficiently inhibit the TNF-α-induced NF-κB signaling pathway.

**Figure 4:**
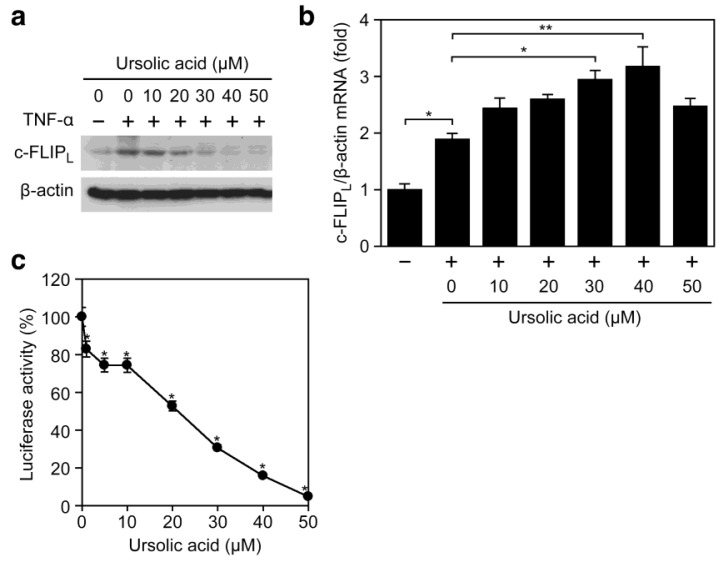
Ursolic acid inhibits TNF-α-induced expression of NF-κB-responsive genes. (**a**) A549 cells were preincubated with various concentrations of ursolic acid for 1 h and then incubated with (+) or without (–) TNF-α (2.5 ng/mL) for 6 h in the presence or absence of ursolic acid. Protein expression of c-FLIP_L_ was analyzed by Western blotting; (**b**) A549 cells were pretreated with indicated concentrations of ursolic acid for 1 h and then incubated with (+) or without (–) TNF-α (2.5 ng/mL) for 6 h in the presence of ursolic acid. c-FLIP_L_ mRNA expression was measured by quantitative RT-PCR. The ratio of c-FLIP_L_ mRNA relative to β-actin mRNA is shown as means ± SD (n = 3). * *P* < 0.05 and ** *P* < 0.01, compared with control; (**c**) A549 cells were transiently transfected with the NF-κB-responsive luciferase reporter for 24 h. The cells were preincubated with various concentrations of ursolic acid for 1 h and then incubated with TNF-α (2.5 ng/mL) for 6 h in the presence of ursolic acid. Cell lysates were prepared and their luciferase activities measured. Luciferase activity (%) is shown as means ± SD (n = 3). * *P* < 0.01, compared with control.

We further addressed whether the inhibitory effect of ursolic acid on TNF-α-induced ICAM-1 expression (Figure 2) is common to other NF-κB-responsive genes and not restricted to ICAM-1. Ursolic acid prevented the TNF-α-induced expression of c-FLIP_L_, a cytosolic protein mainly induced by NF-κB (Figure 4a). By contrast, the TNF-α-induced c-FLIP_L_ mRNA expression was not decreased but rather increased upon treatment with ursolic acid (Figure 4b). Consistently, ursolic acid inhibited the expression of an NF-κB-responsive luciferase reporter to the background level (Figure 4c). Taken together, our present results suggest that ursolic acid inhibits the TNF-α-induced gene expression more effectively by targeting biosynthetic processes from mRNA to protein.

**Figure 5:**
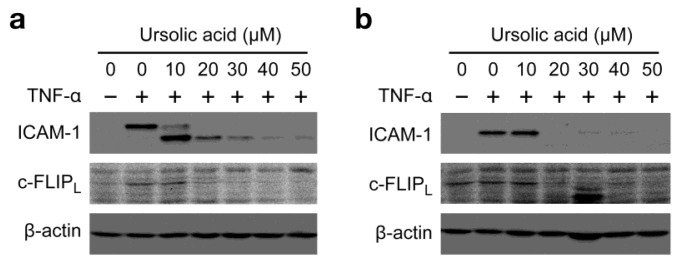
Ursolic acid inhibits TNF-α-induced protein expression of ICAM-1 and c-FLIP_L_. (**a** and **b**) HT-1080 cells (**a**) and MCF7 cells (**b**) were preincubated with various concentrations of ursolic acid for 1 h and then incubated with (+) or without (–) TNF-α (2.5 ng/mL) for 6 h in the presence or absence of ursolic acid. Protein expression of ICAM-1 and c-FLIP_L_ was analyzed by Western blotting.

To broaden the impact of our findings, the biological activity of ursolic acid was examined in two additional cell lines. Human fibrosarcoma HT-1080 cells and human breast adenocarcinoma MCF7 cells were preincubated with ursolic acid for 1 h and then incubated with TNF-α for 6 h in the presence of ursolic acid. As observed in A549 cells, ursolic acid profoundly inhibited the protein expression of ICAM-1 and c-FLIP_L_ at concentrations more than 20 μM (Figure 5a and Figure 5b).

### 3.2. Ursolic Acid Selectively Inhibits Cellular Protein Synthesis

**Figure 6:**
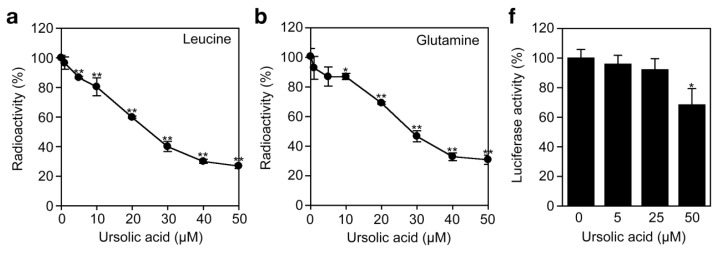

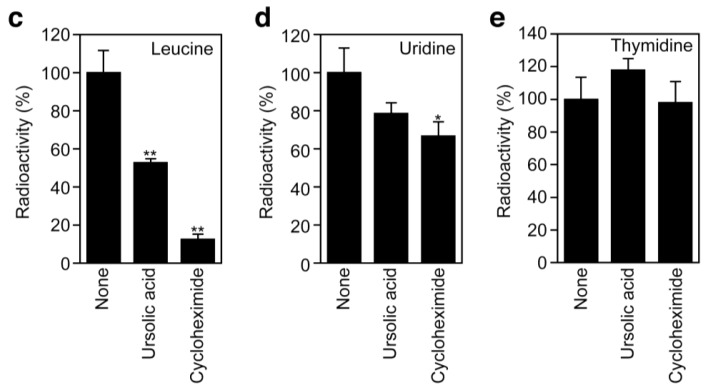
Ursolic acid inhibits cellular protein synthesis. (**a** and **b**) A549 cells were incubated with various concentrations of ursolic acid for 5 h and then incubated with [^3^H]L-leucine (**a**) or [^3^H]L-glutamine (**b**) for 2 h in the presence of ursolic acid. Radioactivity incorporated into the acid-insoluble fractions was measured. Radioactivity (%) is shown as means ± SD (n = 3). * *P* < 0.05 and ** *P* < 0.01, compared with control; (**c** to **e**) A549 cells were preincubated with or without ursolic acid (50 μM) or cycloheximide (10 μM) for 1 h and then incubated with [^3^H]L-leucine (**c**), [^3^H]uridine (**d**), or [^3^H]thymidine (**e**) for 2 h in the presence or absence of those compounds. Radioactivity incorporated into the acid-insoluble fractions was measured. Radioactivity (%) is shown as means ± SD (n = 3). * *P* < 0.05 and ** *P* < 0.01, compared with control; (**f**) Luciferase mRNA was translated by rabbit reticulocyte lysates in the presence of indicated concentrations of ursolic acid at 30 °C for 90 min. Luciferase activity (%) is shown as means ± SD (n = 3). * *P* < 0.01, compared with control.

To investigate the effect of ursolic acid on cellular protein synthesis, A549 cells were labeled with [^3^H]L-amino acids and radioactivity incorporated into the acid-insoluble fractions was measured. Ursolic acid inhibited the incorporation of [^3^H]L-leucine and [^3^H]L-glutamine in a dose-dependent manner (Figure 6a and Figure 6b). Compared with protein synthesis, ursolic acid did not significantly affect the incorporation of [^3^H]thymidine and [^3^H]uridine into DNA and RNA fractions (Figure 6c to Figure 6e). The translation inhibitor cycloheximide exerted an inhibitory profile somewhat different from ursolic acid, as it prevented protein synthesis much more strongly than ursolic acid (Figure 6c). Then, we further investigated the direct effect of ursolic acid on the cell-free translation system where luciferase mRNA was used as a template. Ursolic acid decreased the luciferase activity by 30% at 50 μM (Figure 6f), whereas other translation inhibitors, such as puromycin, prevented luciferase activity completely (data not shown). The mechanism by which ursolic acid partially affects the cell-free translation system is currently unclear. Nevertheless, these data suggest that ursolic acid, unlike cycloheximide or puromycin, directly targets not the translation machinery but the amino acid transport across the plasma membrane.

### 3.3. Ursolic Acid Selectively Inhibits Amino Acid Transport

We have recently shown that the Na^+^/K^+^-ATPase inhibitors, such as ouabain, inhibit cellular protein synthesis by preventing the Na^+^-dependent transport of amino acids in A549 cells [26]. By this mechanism of action, ouabain strongly inhibits TNF-α-induced expression of gene expression at the translational level [26]. To investigate the effect of ursolic acid on the uptake of amino acids into the cell, A549 cells were labeled with [^3^H]L-leucine for 2 h and radioactivity incorporated into the cells was separated into acid-soluble supernatants (free amino acid) and acid-insoluble precipitates (proteins).

**Figure 7:**
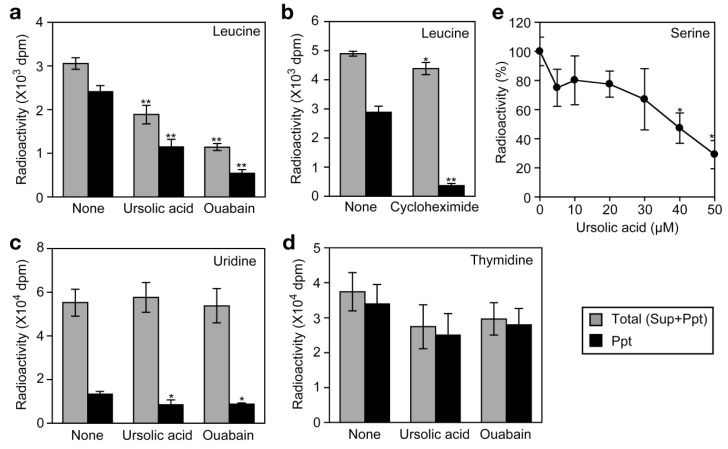
Ursolic acid selectively inhibits the incorporation of amino acids into the cell. (**a** to **d**) A549 cells were preincubated with or without ursolic acid (50 μM) or ouabain (10 μM) for 1 h and then incubated with [^3^H]L-leucine for 2 h in the presence or absence of those two compounds (**a**); A549 cells were preincubated with or without cycloheximide (10 μM) for 1 h and then incubated with [^3^H]L-leucine for 2 h in the presence or absence of cycloheximide (**b**); A549 cells were preincubated with or without ursolic acid (50 μM) or ouabain (10 μM) for 1 h and then incubated with [^3^H]uridine (**c**) or [^3^H]thymidine (**d**) for 2 h in the presence or absence of those two compounds. Radioactivity incorporated into the cell was separated into acid-soluble supernatants (Sup) and acid-insoluble precipitates (Ppt). Total radioactivity incorporated into the cell (Sup+Ppt; gray bars) as well as radioactivity incorporated into the precipitates (Ppt; filled bars) is shown as means ± SD (n = 3). * *P* < 0.05 and ** *P* < 0.01, compared with control; (**e**) A549 cells were preincubated with various concentrations of ursolic acid for 1 h and then pulse-labeled with [^3^H]L-serine for 5 min in the presence of ursolic acid. Radioactivity incorporated into the cell was measured. Data points represent means ± SD (n = 3). * *P* < 0.01, compared with control.

Ouabain strongly decreased the incorporation of [^3^H]L-leucine into the cells as well as into proteins (Figure 7a). By contrast, cycloheximide barely affected the incorporation of [^3^H]L-leucine into the cells but strongly inhibited that into proteins (Figure 7b). Similar to ouabain, ursolic acid decreased the incorporation of [^3^H]L-leucine into the cells as well as into proteins (Figure 7a). The incorporation of [^3^H]uridine and [^3^H]thymidine into the cells was not significantly affected by either ursolic acid or ouabain (Figure 7c and Figure 7d). These data suggest that ursolic acid selectively inhibits the uptake of amino acids into the cells. To investigate the effect of ursolic acid on Na^+^-dependent amino acid transport, A549 cells were preincubated with ursolic acid for 1 h and then pulse-labeled with [^3^H]L-serine, which was shown to be incorporated into A549 cells via a Na^+^-dependent manner [26]. Ursolic acid was found to inhibit the uptake of [^3^H]L-serine (Figure 7e). Thus, these data reveal that ursolic acid inhibits Na^+^-dependent amino acid transport.

### 3.4. Ursolic Acid Directly Inhibits Na^+^/K^+^-ATPase Activity

**Figure 8:**
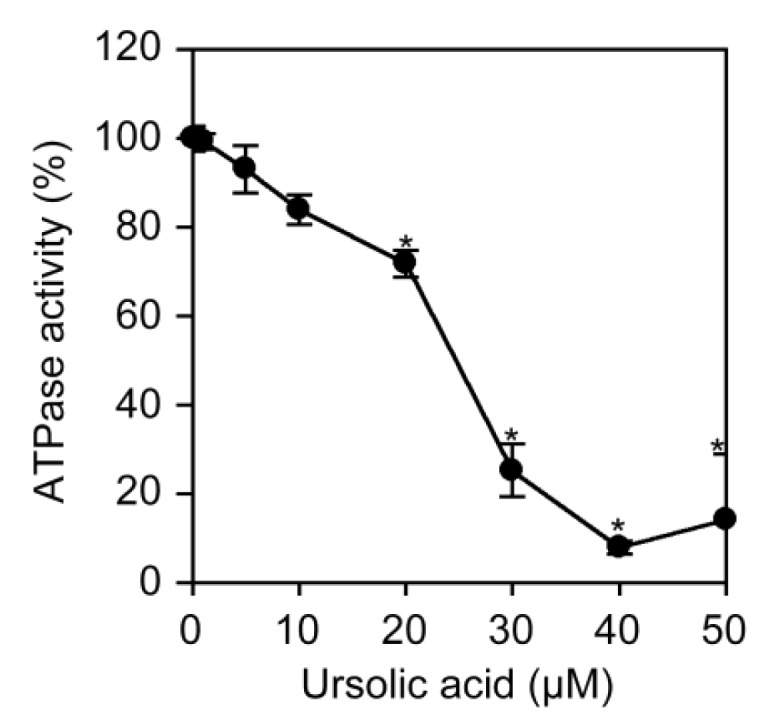
Ursolic acid inhibits Na^+^/K^+^-ATPase activity. Na^+^/K^+^-ATPase highly purified from porcine kidney was preincubated with various concentrations of ursolic acid for 15 min and then incubated with ATP for 30 min in the presence of ursolic acid. The inorganic phosphates produced by ATP hydrolysis were measured. Data points represent means ± SD (n = 3). * *P* < 0.01, compared with control.

Na^+^/K^+^-ATPase pumps Na^+^ out and K^+^ in via the hydrolysis of ATP and plays a major role in maintaining Na^+^ and K^+^ gradients that couple amino acid transport. To investigate the direct effect of ursolic acid on Na^+^/K^+^-ATPase activity, Na^+^/K^+^-ATPase highly purified from porcine kidney was incubated in the presence of ursolic acid and ATP, and this was followed by measurement of inorganic phosphates released by hydrolysis. Ursolic acid inhibited ATPase activity in a dose-dependent manner and at the IC_50_ value of 24.7 μM (Figure 8).

### 3.5. The Biological Properties of Ursolic Acid

Pro-inflammatory cytokines activate the NF-κB signaling pathway and induce the expression of a wide range of NF-κB-responsive genes, such as ICAM-1. In our screening for anti-inflammatory agents, ursolic acid was initially identified to inhibit cell-surface ICAM-1 expression induced by pro-inflammatory cytokines, such as TNF-α [9,10]. It has been reported that ursolic acid inhibits the NF-κB signaling pathway constitutively activated or induced by pro-inflammatory cytokines or chemotherapeutic agents [11,12,13,14,15,16,17,18]. However, in contrast to these previous reports, we found that ursolic acid only partially diminishes TNF-α-induced NF-κB signaling pathway but more effectively inhibits the TNF-α-induced NF-κB-responsive gene expression by targeting cellular protein synthesis at least in A549 cells. The ability of ursolic acid to inhibit the NF-κB signaling pathway may be cell-type-specific or influenced by experimental conditions such as incubation periods. In fact, relatively long pre-incubation periods (8 to 48 h) with ursolic acid (10 to 100 μM) were used to block constitutive and inducible NF-κB activation in human cancer cell lines including A549 cells [11,12,15,18].

Cardiac glycosides, such as ouabain, are known to be highly specific inhibitors of Na^+^/K^+^-ATPase activity. We have recently shown that ouabain and odoroside A inhibit the ATP-hydrolyzing activity of porcine kidney Na^+^/K^+^-ATPase at the IC_50_ values of 1.6 μM and 1.2 μM, respectively [26]. Using the same experimental systems, ursolic acid was found to inhibit Na^+^/K^+^-ATPase activity at the IC_50_ value of 24.7 μM. Thus, ursolic acid can be regarded as an inhibitor of Na^+^/K^+^-ATPase activity with 10- to 20-fold weaker potency than cardiac glycosides. In agreement with this, it has been recently shown that ursolic acid inhibits the activity of Na^+^/K^+^-ATPase purified from cerebral cortex at the IC_50_ value of 76.7 μM [27] as well as Na^+^/K^+^-ATPase activity in the crude mitochondrial fractions of human cell lines [28]. Moreover, a theoretical modeling study shows that ouabain and ursolic acid bind to Na^+^/K^+^-ATPase in a distinct manner [27,29]. In agreement with our previous study using cardiac glycosides [26], our present study for the first time reveals a link between the inhibition of Na^+^/K^+^-ATPase activity by ursolic acid and the prevention of NF-κB-inducible gene expression at the post-transcriptional level.

Membrane transport of amino acids in mammalian cells is accomplished by a number of discrete systems and has distinct substrate specificity [30]. As a primary transporter, Na^+^/K^+^-ATPase pumps Na^+^ out and K^+^ in by hydrolyzing ATP and maintains Na^+^ and K^+^ gradients across plasma membranes [31]. Some secondary amino acid transporters are coupled with electrical and chemical gradients by primary active transporters [30]. We have recently shown that ouabain selectively inhibits both Na^+^-dependent membrane transport of amino acids and cellular protein synthesis by blocking Na^+^/K^+^-ATPase activity [26]. In accord with this notion, ursolic acid was found to inhibit the Na^+^-dependent membrane transport of L -serine as well as to diminish cellular protein synthesis much more strongly than DNA/RNA syntheses. Therefore, it seems most likely that ursolic acid mainly inhibits cellular protein synthesis by targeting Na^+^/K^+^-ATPase in the same mechanism as ouabain. However, as described previously [11,12,13,14,15,16,17,18], it should be noted that ursolic acid may interfere with intracellular proteins other than Na^+^/K^+^-ATPase as primary targets and modulate the NF-κB signaling pathway in different experimental settings.

## 4. Conclusions

Our present study provides a novel molecular mechanism in which ursolic acid inhibits the translation process during the TNF-α-induced NF-κB-dependent gene expression by preventing Na^+^/K^+^-ATPase. Pro-inflammatory cytokines and their activation of the NF-κB signaling pathway play a critical role in chronic inflammation, which is known to be associated with autoimmune diseases, cancer, and metabolic disorders. Ursolic acid is a constituent of popular herbs and foods. Thus, it seems plausible that ursolic acid is a beneficial natural compound that exerts anti-inflammatory activity and is effective for the prevention of chronic inflammation.

## References

[1] Collins T., Read M.A., Neish A.S., Whitley M.Z., Thanos D., Maniatis T. (1995). Transcriptional regulation of endothelial cell adhesion molecules: NF-κB and cytokine-inducible enhancers. FASEB J..

[2] Cook-Mills J.M., Deem T.L. (2005). Active participation of endothelial cells in inflammation. J. Leukoc. Biol..

[3] Roebuck K.A., Finnegan A. (1999). Regulation of intercellular adhesion molecule-1 (CD54) gene expression. J. Leukoc. Biol..

[4] Hayden M.S., Ghosh S. (2008). Shared principles in NF-κB signaling. Cell.

[5] Bhoj V.G., Chen Z.J. (2009). Ubiquitylation in innate and adaptive immunity. Nature.

[6] Baud V., Karin M. (2009). Is NF-κB a good target for cancer therapy? Hopes and pitfalls. Nat. Rev. Drug Discov..

[7] Kataoka T. (2009). Chemical biology of inflammatory cytokine signaling. J. Antibiot..

[8] Ikeda Y., Murakami A., Ohigashi H. (2008). Ursolic acid: An anti- and pro-inflammatory triterpenoid. Mol. Nutr. Food Res..

[9] Fu L., Zhang S., Li N., Wang J., Zhao M., Sakai J., Hasegawa T., Mitsui T., Kataoka T., Oka S. (2005). Three new triterpenes from *Nerium oleander* and biological activity of the isolated compounds. J. Nat. Prod..

[10] Zhao M., Zhang S., Fu L., Li N., Bai J., Sakai J., Wang L., Tang W., Hasegawa T., Ogura H. (2006). Taraxasterane- and ursane-type triterpenes from *Nerium oleander* and their biological activities. J. Nat. Prod..

[11] Shishodia S., Majumdar S., Banerjee S., Aggarwal B.B. (2003). Ursolic acid inhibits nuclear factor-KB activation induced by carcinogenic agents through suppression of IκBα kinase and p65 phosphorylation: Correlation with down-regulation of cyclooxygenase 2, matrix metalloproteinase 9, and cyclin D1. Cancer Res..

[12] Hsu Y.L., Kuo P.L., Lin C.C. (2004). Proliferative inhibition, cell-cycle dysregulation, and induction of apoptosis by ursolic acid in human non-small cell lung cancer A549 cells. Life Sci..

[13] Manu K.A., Kuttan G. (2008). Ursolic acid induces apoptosis by activating p53 and caspase-3 gene expressions and suppressing NF-κB mediated activation of bcl-2 in B16F-10 melanoma cells. Inter. Immunopharmacol..

[14] Huang H.C., Huang C.Y., Lin-Shiau S.Y., Lin J.K. (2009). Ursolic acid inhibits IL-1β or TNF-α-induced C6 glioma invasion through suppressing the association ZIP/p62 with PKC-ζ and downregulating the MMP-9 expression. Mol. Carcinog..

[15] Li Y., Xing D., Chen Q., Chen W.R. (2010). Enhancement of chemotherapeutic agent-induced apoptosis by inhibition of NF-κB using ursolic acid. Inter. J. Cancer.

[16] Shyu M.H., Kao T.C., Yen G.C. (2010). Oleanoic acid and ursolic acid induce apoptosis in HuH7 human hepatocellular carcinoma cells through a mitochondrial-dependent pathway and downregulation of XIAP. J. Agric. Food Chem..

[17] Takada K., Nakane T., Masuda K., Ishii H. (2010). Ursolic acid and oleanolic acid, members of pentacyclic triterpenoid acids, suppress TNF-α-induced E-selectin expression by cultured umbilical vein endothelial cells. Phytomedicine.

[18] Yeh C.T., Wu C.H., Yen G.C. (2010). Ursolic acid, a naturally occurring triterpenoid, suppresses migration and invasion of human breast cancer cells by modulating c-Jun *N*-terminal kinase, Akt and mammalian target of rapamycin signaling. Mol. Nutr. Food Res..

[19] Utreras E., Ossandon P., Acuña-Castillo C., Varela-Nallar L., Müller C, Arraztoa J.A, Cardenas H., Imarai M. (2000). Expression of intercellular adhesion molecule 1 (ICAM-1) on the human oviductral epithelium and mediation of lymphoid cell adherence. J. Reprod. Fertil..

[20] Wang S., Zhang Z.X., Yin Z., Liu W., Garcia B., Huang X., Acott P., Jevnikar A.M. (2011). Anti-IL-2 receptor antibody decreases cytokine-induced apoptosis of human renal tubular epithelial cells (TEC). Nephrol. Dial. Transplant..

[21] Hayashi Y., Mimura K., Matsui H., Takagi T. (1989). Minimum enzyme unit for Na^+^/K^+^-ATPase is the αβ-protomer. Determination by low-angle laser light scattering photometry coupled with high-performance gel chromatography for substantially simultaneous measurement of ATPase activity and molecular weight. Biochim. Biophys. Acta.

[22] Kobayashi T., Tahara Y., Takenaka H., Mimura K., Hayashi Y. (2007). Na^+^- and K^+^-dependent oligomeric interconversion among αβ-protomers, diprotomers and higher oligomers in solubilized Na^+^/K^+^-ATPase. J. Biochem..

[23] Takada Y., Matsuo K., Kataoka T. (2008). Gramicidin A directly inhibits mammalian Na^+^/K^+^-ATPase. Mol. Cell. Biochem..

[24] Hoenig M., Lee R.J., Ferguson D.C. (1989). A microtiter plate assay for inorganic phosphate. J. Biochem. Biophys. Methods.

[25] Holden N.S., Catley M.C., Cambridge L.M., Barnes P.J., Newton R. (2004). ICAM-1 expression is highly NF-κB-dependent in A549 cells. No role for ERK and p38 MAPK. Eur. J. Biochem..

[26] Takada Y., Matsuo K., Ogura H., Bai L., Toki A., Wang L., Ando M., Kataoka T. (2009). Odoroside A and ouabain inhibit Na^+^/K^+^-ATPase and prevent NF-κB-inducible protein expression by blocking Na^+^-dependent amino acid transport. Biochem. Pharmacol..

[27] Chen R.J., Chung T.Y., Li F.Y., Yang W.H., Jinn T.R., Tzen J.T. (2010). Steroid-like compounds in Chinese medicines promote blood circulation via inhibition of Na^+^/K^+^-ATPase. Acta Pharmacol. Sin..

[28] Yan S.L., Huang C.Y., Wu S.T., Yin M.C. (2010). Oleanoic acid and ursolic acid induce apoptosis in four human liver cancer cell lines. Toxicol. in Vitro.

[29] Chen R.J., Jinn T.R., Chen Y.C., Chung T.Y., Yang W.H., Tzen J.T. (2011). Active ingredients in Chinese medicines promoting blood circulation as Na^+^/K^+^-ATPase inhibitors. Acta Pharmacol. Sin..

[30] Hyde R., Taylor P.M., Hundal H.S. (2003). Amino acid transporters: Roles in amino acid sensing and signalling in animal cells. Biochem. J..

[31] Kaplan J.H. (2002). Biochemistry of Na, K-ATPase. Annu. Rev. Biochem..

